# Genetic variations of TLR5 gene interacted with Helicobacter pylori infection among carcinogenesis of gastric cancer

**DOI:** 10.18632/oncotarget.16050

**Published:** 2017-03-09

**Authors:** Tianwen Xu, Deqiang Fu, Yi Ren, Yijun Dai, Jianguang Lin, Liming Tang, Jian Ji

**Affiliations:** ^1^ Department of Oncology, The Second Affiliated Hospital of Fujian Medical University, China; ^2^ Department of Thyroid and Breast, Huai'an First People's Hospital, Nanjing Medical University, China; ^3^ No. 2 People's Hospital of Henan Province, China; ^4^ Department of Thoracic Surgery, Huai’an First People's Hospital, Nanjing Medical University, China

**Keywords:** gastric cancer, variation, genetic, TLR5, helicobacter pylori

## Abstract

Gastric cancer (GC) ranks the second prevalent cancer type and the second cancer-related death in China. However, the precise mechanisms of GC development remain poorly understood. Chronic infection with Helicobacter pylori is the strongest identified risk factor for GC. Toll-like receptor (TLR) genes, which play critical roles in Helicobacter pylori induced chronic inflammation, may also be implicated in GC susceptibility. TLR5 signaling deficiency could deregulate a cascade of inflammatory events. In current study, we systematically evaluated genetic variations of TLR5, and their interaction with Helicobacter pylori infection among carcinogenesis of gastric cancer, using a large case-controls study among Chinese population. Minor alleles of three SNPS, including rs5744174 (*P* = 0.001), rs1640827 (*P* = 0.005), and rs17163737 (*P* = 0.004), were significantly associated with increased GC risk (OR ranged from 1.20–1.24). Significant interactions with Helicobacter pylori infection were also identified for rs1640827 (P for interaction = 0.009) and rs17163737 (P for interaction = 0.006). These findings suggest that genetic variants in TLR5 may modify the role of Helicobacter pylori infection in the process of causing GC.

## INTRODUCTION

Gastric cancer (GC), the second frequent cause of cancer-related deaths worldwide, remains one of the major problem of public health [1]. Its incidence rates are highest in Eastern Asia, particularly in China, Korea, Mongolia, and Japan [2]. According to the program of surveillance and health services research of American Cancer Society, 26,370 estimated new GC cases and 10,730 deaths were diagnosed in USA in 2016, during which approximately twice as high in men as in women and vary widely across States [3]. In China, the situation that GC ranks the second prevalent cancer type and the second cancer-related death should drew more attention [4]. According to the National Office for Cancer Prevention and Control of China (NOCPCC), 679.1 thousands new GC cases and 498.0 deaths were found in China in 2015 [4]. Chronic infection with Helicobacter pylori, accounting about 90% of new cases of noncardia gastric cancer worldwide, is the strongest identified risk factor for GC development [2, 4–11]. Despite accumulating evidence indicating the involvement of multiple gene–environment interactions with Helicobacter pylori infection, the precise mechanisms of GC development remain poorly understood.

Genetic polymorphisms in inflammatory response-related genes has been identified to contribute to susceptibility of GC and its precursors [12, 13]. Toll-like receptor (TLR) genes, which play critical roles in Helicobacter pylori induced chronic inflammation, may also be implicated in GC susceptibility [14–16]. TLR5 signaling deficiency could change interactions with commensal microbiota and deregulate a cascade of inflammatory events that can suppress or accelerate extra-intestinal cancers [17, 18]. Functional TLR5 genetic variants could also affect human colorectal cancer survival [19]. Here, we systematically evaluated the function of genetic variations of TLR5, and their interaction with Helicobacter pylori infection among carcinogenesis of gastric cancer in a Chinese population.

## RESULTS

### Basic characteristics of study subjects

Totally included in this study were 1,300 GC cases and 1,300 healthy controls. Detailed information on the basic characteristics of the study population is presented in Table 1. There were no significant differences in age, gender, education level, body mass index, and drinking status between the GC patients and the controls. However, significant difference was detected for the distributions of residence (*P* = 0.028), smoking status (*P* < 0.001), and Helicobacter pylori infection (*P* < 0.001) between the two groups, which indicates rural residence, smoking, and Helicobacter pylori infection are risk factors for the susceptibility of GC.

**Table 1:** Distributions of select variables in GC patients and cancer-free controls

Characteristics	Cases	Controls	*P* values
Number	1300	1300	
Age (mean ± SD)	59.99 ± 10.56	59.35 ± 9.76	0.109
Gender			
Male	918 (70.6%)	933 (71.8%)	0.496
Female	382 (29.4%)	367 (28.2%)	
Education level			
≥ Middle school	270 (20.8%)	295 (22.7%)	0.235
< Middle school	1030 (79.2%)	1005 (77.3%)	
Residence, (*n* (%))			
Rural	690 (53.1%)	634 (48.8%)	**0.028**
Urban	610 (46.9%)	666 (51.2%)	
Body mass index	22.2 ± 3.1	22.4 ± 3.5	0.123
Smoking status			
Yes	400 (30.8%)	265 (20.4%)	**< 0.001**
No	900 (69.2%)	1035 (79.6%)	
Drinking status			
Yes	367 (28.2%)	325 (25.0%)	0.062
No	933 (71.8%)	975 (75.0%)	
Helicobacter pylori infection			
Yes	800 (61.5%)	586 (45.1%)	**< 0.001**
No	500 (38.5%)	714 (54.9%)	

### Association between TLR5 gene polymorphisms and risk of GC

In current study, 7 tagSNPs of TLR5 gene, including rs5744174, rs5744140, rs5744113, rs1640827, rs2241096, rs17163737, and rs2241097, were evaluated for their association with susceptibility of GC (Table 2). All the seven SNPs did not depart from the Hardy–Weinberg equilibrium in the healthy controls (*p* > 0.05). As shown in Table 3, we found minor alleles of rs5744174 (*P* = 0.001), rs1640827 (*P* = 0.005), and rs17163737 (*P* = 0.004) were significantly associated with increased GC risk (OR ranged from 1.20–1.24). Even adjusted for Bonferroni correction, the results were still significant (*P* = 0.001*7=0.007; *P* = 0.005*7=0.035; *P* = 0.004*7=0.028). For rs5744174, carriers of genotype TC (OR = 1.18; 95%=1.00–1.39; *P* = 0.047) and CC (OR = 1.68; 95%=1.18–2.41; *P* = 0.004) have a higher GC risk, compared with carriers of genotype TT. For rs1640827, carriers of genotype TC (OR = 1.19; 95%=1.00–1.41; *P* = 0.048) and CC (OR = 1.69; 95%=1.08–2.62; *P* = 0.021) have a higher GC risk, compared with carriers of genotype TT. For rs17163737, carriers of genotype GT (OR = 1.19; 95%=1.01–1.40; *P* = 0.034) and TT (OR = 1.41; 95%=1.05–1.90; *P* = 0.023) have a higher GC risk, compared with carriers of genotype GG. We didn't detected any significant associations for including rs5744140, rs5744113, rs2241096, and rs2241097. Sensitivity analyses were also conducted by including other non-significant variables in Table 1, however, the results were not changed (data not shown).

**Table 2:** TagSNP selection of TLR5 gene

TagSNP	Size	ave.MAF	ave.r2	SNPs.captured
rs5744174	8	0.1969	0.911	<CHB> rs1100886,rs5744174,rs851139,rs851178,rs851180,rs851186,rs851192,rs851193
rs5744140	6	0.083	1	<CHB> rs5744135,rs5744138,rs5744139,rs5744140,rs5744143,rs5744149
rs5744113	5	0.2548	0.9538	<CHB> rs1341987,rs2096141,rs2096142,rs2353476,rs5744113
rs1640827	2	0.161	1	<CHB> rs1640827,rs851191
rs2241096	1	0.311	1	<CHB> rs2241096
rs17163737	1	0.286	1	<CHB> rs17163737
rs2241097	1	0.137	1	<CHB> rs2241097

**Table 3:** Association between TLR5 gene polymorphisms and risk of GC

Genotype	Cases	Controls	OR (95% CI)*	*P* value
**rs5744174**				
TT	794	858	1.00 (reference)	
TC	425	390	1.18 (1.00–1.39)	**0.047**
CC	81	52	1.68 (1.18–2.41)	**0.004**
T			1.00 (reference)	
C			1.24 (1.09–1.42)	**0.001**
**rs5744140**				
CC	1069	1098	1.00 (reference)	
CT	212	189	1.15 (0.93–1.43)	0.193
TT	19	13	1.50 (0.74–3.04)	0.259
C			1.00 (reference)	
T			1.18 (0.97–1.43)	0.089
**rs5744113**				
AA	768	772	1.00 (reference)	
AG	440	448	0.99 (0.84–1.16)	0.879
GG	92	80	1.15 (0.84–1.58)	0.368
A			1.00 (reference)	
G			1.03 (0.91–1.17)	0.602
**rs1640827**				
TT	842	901	1.00 (reference)	
TC	406	366	1.19 (1.00–1.41)	**0.048**
CC	52	33	1.69 (1.08–2.62)	**0.021**
T			1.00 (reference)	
C			1.22 (1.06–1.41)	**0.005**
**rs2241096**				
GG	762	772	1.00 (reference)	
AG	471	456	1.05 (0.89–1.23)	0.585
AA	67	52	1.31 (0.89–1.90)	0.164
G			1.00 (reference)	
A			1.08 (0.95–1.23)	0.231
**rs17163737**				
GG	679	745	1.00 (reference)	
GT	509	468	1.19 (1.01–1.40)	**0.034**
TT	112	87	1.41 (1.05–1.90)	**0.023**
G			1.00 (reference)	
T			1.20 (1.06–1.35)	**0.004**
**rs2241097**				
AA	974	990	1.00 (reference)	
AC	307	296	1.05 (0.88–1.26)	0.571
CC	19	14	1.38 (0.69–2.76)	0.363
A			1.00 (reference)	
C			1.07 (0.91–1.26)	0.384

*Adjusted for residence, smoking status and Helicobacter pylori infection

### Interaction analyses between TLR5 gene polymorphisms and Helicobacter pylori infection

We also evaluated the joint effects of TLR5 gene polymorphisms and Helicobacter pylori infection on GC (Table 4). Significant interactions with Helicobacter pylori infection were identified for rs1640827 (P for interaction = 0.009) and rs17163737 (P for interaction = 0.006). For SNP rs1640827, we found 2.13-fold elevated GC risk for subjects with genotype TC+CC and with Helicobacter pylori infection (OR = 2.13, 95% CI: 1.79–2.53). While for SNP rs17163737, we found 2.17-fold elevated GC risk for subjects with genotype GT+TT and with Helicobacter pylori infection (OR = 2.17, 95% CI: 1.81–2.61). However, no statistically significant interaction between rs5744174 and Helicobacter pylori infection was found.

**Table 4:** Interaction analyses between TLR5 gene polymorphisms and Helicobacter pylori infection

	Helicobacter pylori infection					
	No			Yes		
	Case	Control	OR(95% CI)*	Case	Control	OR(95% CI)*
rs5744174						
TT	285	467	1.00 (reference)	509	391	2.13 (1.75–2.60)
TC+CC	215	247	1.43 (1.13–1.80)	291	195	2.23 (1.87–2.68)
	**P for interaction = 0.114**					
rs1640827						
TT	330	515	1.00 (reference)	512	386	2.07 (1.71–2.50)
TC+CC	170	199	1.33 (1.04–1.71)	288	200	2.13 (1.79–2.53)
	**P for interaction = 0.009**					
rs17163737						
GG	270	430	1.00 (reference)	409	315	2.07 (1.67–2.55)
GT+TT	230	284	1.29 (1.02–1.62)	391	271	2.17 (1.81–2.61)
	**P for interaction = 0.006**					

*Adjusted for residence, and smoking status

## DISCUSSION

The current study systematically explored the function of genetic variations of TLR5, and their interaction with Helicobacter pylori infection among carcinogenesis of gastric cancer in a Chinese population, using a large scale, case-control study. TLR5, which could recognize bacterial flagellin from invading mobile bacteria, has an close relationship with Helicobacter pylori infection [20–22]. We found rs5744174, rs1640827 and rs17163737 were significantly associated with increased GC risk. Also, significant interactions with Helicobacter pylori infection were also identified for rs1640827 and rs17163737. To our best knowledge, this should be the largest, and most comprehensive study to systematically explore genetic variations of TLR5, and their interaction with Helicobacter pylori infection among carcinogenesis of gastric cancer.

GC remains a major public health problem and disease burden in China. Infection of Helicobacter pylori, a Gram-negative, microaerophilic, flagellated bacteria that adheres to human gastric mucosa, has been identified to increase GC risk for decades [23]. TLR5 recognizes flagellin, which is the protein monomer that makes up the filament of bacterial flagella, and found on most motile bacteria [24]. Then, Smith et al [25] identified TLR5 was required for Helicobacter pylori-induced NF-kappa B activation and chemokine expression by epithelial cells, and demonstrated that gastric epithelial cells recognized and responded to H. pylori infection via TLR5, which indicated the close relationship between TLR5 and Helicobacter pylori infection. TLR5 is located at 1q41, containing 6 exons. Abnormal TLR5 functioning is related to the onset of gastric, cervical, endometrial and ovarian cancers [26, 27]. In Trejo-de's paper [28], they found that Patients with polymorphisms of TLR5 expressed significantly lower levels of IL-1b, TNF-a, IL-6 and IL-10 in gastric tissue. Here, we found TLR5 rs5744174, rs1640827 and rs17163737 were significantly associated with increased GC risk. One previous study found TLR5 rs5744174, and interaction between rs5744174 and H. pylori infection were associated with the development of GC with a smaller sample size [13]. However, we didn't replicate the infection in current study, although we found the significant association between TLR5 rs5744174 and GC risk. Recently, TLR5 rs5744174 was also identified to be significantly associated with HDL-C (recessive model: β = –0.14, 95 % CI: –0.24 to –0.03, *P* = 0.009) [29]. Significant differences were observed in the distribution of alleles and genotypes between the patients with rheumatic heart disease (RHD) and the controls for rs1640827 [30].

Strengths of this study included the large sample size which could provide enough statistical power (the power for rs5744174, rs1640827 and rs17163737 were 88.1%, 78.6%, and 82.6%, respectively), the high participation rate, and the homogeneous ethnic background of the participants. Also, some limitations should also be acknowledged in the present study. First, the study population was limited to the Chinese population; second, selection bias may emerge during the recruitment of study percipients, due to the hospital-based study design. Further, results derived from other populations, and association studies between the TLR5 polymorphisms and expression of inflammatory cytokines in the gastric cancer tissues are needed to better understand the complicated mechanisms underlying the modifying effect of the TLR5 gene.

In summary, we identified significant interactions between TLR5 rs1640827, rs17163737 and Helicobacter pylori infection. This results provide valuable insights into the TLR5 and Helicobacter pylori infection involved in gastric carcinogenesis, and this may have important implications in personalized prevention of GC.

## MATERIALS AND METHODS

### Study subjects

In current study, all cases were confirmed by examination of gastroscopy biopsy or surgical specimens, while the age, gender, and education level matched controls were randomly enrolled during the same time period, and were confirmed to have no current or previous signs of cancer. Information on age, gender, education levels, residence, smoking status, drinking status, and body mass index were obtained by questionnaires. A 5-mL blood sample was collected from each subject. All subjects gave written informed consent for this study, and this study was approved by the appropriate Ethics Committee.

### DNA extraction and genotyping

Genomic DNA was isolated from peripheral blood using the QIAamp DNA Blood Maxi Kit (Qiagen, Hilden, Germany) according to the manufacturer's protocol. TagSNPs of TLR5 based on SNPs with minor allele frequencies (MAF) greater than 0.05 in CHB (Han Chinese in Beijing, China) population were selected using the web-based software SNPinfo (http://snpinfo.niehs.nih.gov/), which produced 7 tagSNPs (Table 2). Genotyping were conducted using Sequenom MassARRAY platform, while Sequenom Typer 4.0 software (Sequenom) was used for data management and analyses. Randomly selected 5% of samples were run in duplicates and the concordance of genotype calls was 100% for duplicate samples. All laboratory personnel were blinded to the case-control status of the study subjects.

### Determination of Helicobacter pylori infection

Sero-status of antibodies to four H. pylori specific antigens (CagA, VacA, UreA and UreB) was determined using Typing Detection Kit for Antibody to H. pylori (Shenzhen Blot Biotech Co., Ltd, Shenzhen, China) according to the manufacturer's instructions. H. pylori sero-positivity was defined as any of the CagA, VacA, UreA or UreB being seropositive.

### Statistical analysis

All the statistical analysis was performed by SPSS 19.0 software (IBM Corporation, Armonk, NY, USA). All statistical tests were two-tailed, and a *P-value* < 0.05 was considered statistically significant. Hardy-Weinberg equilibrium (HWE) was assessed by Pearson's goodness-of-fit Chi-square (χ2) statistic for each SNP. The differences of qualitative characteristics were analyzed using the χ2 test, while continuous variables were expressed as the mean ±standard deviation (SD), and were tested by the student's t test. Odds ratios (ORs) with 95% confidence intervals (CIs) were calculated for each allele in both the patient and control groups, using a binary logistic regressive analysis by adjusting for potential confounding factors. A likelihood ratio test was conducted by comparing the model including only the main effects with that including both the main effects and the interaction terms to derive the *P value* for the multiplicative interaction test.
